# Supplementary material for: Analysis of Food Pairing in Regional Cuisines of India
Source: PLoS One. 2015 Oct 2;10(10):e0139539. doi: 10.1371/journal.pone.0139539 (PMC4592201; doi:10.1371/journal.pone.0139539)
Supplement: S1 Table — Number of ingredients in each category for all regional cuisines. (PDF) [file pone.0139539.s001.pdf]

Supporting Information

S1 Table Distribution of ingredients across categories

Ingredient Category	Bengali	Gujarati	Jain	Maharashtrian	Mughlai	Punjabi	Rajasthani	South Indian
**spice**	25	23	26	25	24	33	21	25
**vegetable**	14	23	29	14	15	29	16	23
**fruit**	13	19	25	9	16	22	5	14
**plant derivative**	8	7	11	7	8	13	4	6
**nut/seed**	12	12	12	11	11	13	8	10
**cereal/crop**	6	10	11	6	9	12	7	9
**dairy**	7	6	8	6	7	10	5	7
**plant**	2	3	3	3	4	5	4	5
**pulse**	4	6	5	4	5	6	5	6
**herb**	2	2	5	3	3	4	2	3
**meat**	3	0	0	2	0	1	0	0
**beverage**	1	0	1	1	0	1	0	0
**fish/seafood**	2	0	0	0	0	0	0	2
**animalproduct**	2	0	1	1	2	2	0	2
**flower**	1	1	1	1	1	1	1	1
**additive**	0	0	0	0	0	0	0	1

Table 1: Number of ingredients in each category for all regional cuisines.
