# Supplementary material for: Analysis of Food Pairing in Regional Cuisines of India
Source: PLoS One. 2015 Oct 2;10(10):e0139539. doi: 10.1371/journal.pone.0139539 (PMC4592201; doi:10.1371/journal.pone.0139539)
Supplement: S2 Table — Exponents (α) for regional cuisines and their random controls. (PDF) [file pone.0139539.s002.pdf]

Supporting Information

S2 Table Exponents (α) of Sigmoid fits for $P(N_s^R)$ vs N_s^R distribution

Cuisine	α Values				
	Original	R0	R1	R2	R3
**Bengali**	0.255525	0.181436	0.255149	0.190506	0.26209
**Gujarati**	0.405862	0.187475	0.365109	0.207978	0.37633
**Jain**	0.226656	0.155991	0.235283	0.138507	0.228731
**Maharashtrian**	0.282265	0.158809	0.259422	0.141178	0.269226
**Mughlai**	0.184891	0.173672	0.202563	0.143178	0.194965
**Punjabi**	0.207118	0.150068	0.207771	0.120212	0.215736
**Rajasthani**	0.315478	0.223507	0.35912	0.209513	0.351726
**South Indian**	0.300892	0.189509	0.280907	0.213137	0.290387

Table 1: Exponents (α) for regional cuisines and their random controls.
