# Supplementary material for: Analysis of Food Pairing in Regional Cuisines of India
Source: PLoS One. 2015 Oct 2;10(10):e0139539. doi: 10.1371/journal.pone.0139539 (PMC4592201; doi:10.1371/journal.pone.0139539)
Supplement: S3 Table — Power law exponents (γ) of all regional cuisines. (PDF) [file pone.0139539.s003.pdf]

Supporting Information

S3 Table Power law exponents (γ) for $f(N)$ vs N distribution

Cuisine	γ Values
**Bengali**	1.71906
**Gujarati**	2.11136
**Jain**	1.77156
**Maharashtrian**	1.6974
**Mughlai**	1.47354
**Punjabi**	1.55844
**Rajasthani**	2.62489
**South Indian**	1.948

Table 1: Power law exponents (γ) of all regional cuisines.
