# Supplementary material for: Analysis of Food Pairing in Regional Cuisines of India
Source: PLoS One. 2015 Oct 2;10(10):e0139539. doi: 10.1371/journal.pone.0139539 (PMC4592201; doi:10.1371/journal.pone.0139539)
Supplement: S4 Table — Details of top 10 ingredients contributing to positive and negative food pairing in each of the regional cuisines. (PDF) [file pone.0139539.s004.pdf]

Supporting Information

S4 Table Ingredients contributing significantly to food pairing

Bengali					
**Ingredients contributing to negative food pairing**	**χ value**	**Frequency of occurrence**	**Ingredients contributing to positive food pairing**	**χ value**	**Frequency of occurrence**
coriander	-0.24319	40	milk	0.84165	31
ginger garlic paste	-0.21437	16	cottage cheese	0.38636	11
garam masala	-0.20126	14	orange	0.21789	4
mango	-0.19701	13	buttermilk	0.17259	25
cayenne	-0.13469	65	coconut	0.13006	12
tomato	-0.11413	14	rose	0.12178	5
tamarind	-0.11053	9	cocoa	0.08218	5
green bell pepper	-0.10233	26	strawberry	0.05512	2
cumin	-0.06875	36	cream	0.05368	5
mung bean	-0.06702	4	saffron	0.05329	14
**Gujarati**					
**Ingredients contributing to negative food pairing**	**χ value**	**Frequency of occurrence**	**Ingredients contributing to positive food pairing**	**χ value**	**Frequency of occurrence**
green bell pepper	-0.29066	169	cardamom	0.17035	43
cayenne	-0.19164	145	milk	0.158002	34
mung bean	-0.09783	37	mango	0.15628	20
coriander	-0.05721	45	lemon	0.11942	31
garam masala	-0.05695	26	strawberry	0.07485	2
black pepper	-0.05281	33	chaat masala	0.06775	4
asafoetida	-0.04863	169	apple	0.06058	2
coriander cumin seeds powder	-0.04469	26	mint	0.05999	11
sesame seed	-0.04148	62	apricot	0.05948	1
turmeric	-0.03435	157	cottage cheese	0.05743	4
**Jain**					
**Ingredients contributing to negative food pairing**	**χ value**	**Frequency of occurrence**	**Ingredients contributing to positive food pairing**	**χ value**	**Frequency of occurrence**
cayenne	-0.18622	152	butter	1.22722	68
garam masala	-0.14199	28	milk	0.85545	62
mango	-0.11421	24	bread	0.26881	25
black bean	-0.08291	33	corn	0.26018	29
coriander	-0.06855	47	cocoa	0.14714	3
tamarind	-0.06793	17	cream	0.11764	37
black pepper	-0.06234	55	peanut butter	0.09925	4
green bell pepper	-0.06095	112	grape	0.09078	4
ginger	-0.06059	17	cheese	0.08762	11
chaat masala	-0.05613	14	strawberry	0.08254	4
**Maharashtrian**					
**Ingredients contributing to negative food pairing**	**χ value**	**Frequency of occurrence**	**Ingredients contributing to positive food pairing**	**χ value**	**Frequency of occurrence**
cayenne	-0.20961	71	strawberry	0.18767	1
green bell pepper	-0.16631	27	apricot	0.17937	1

cardamom	-0.13171	28	milk	0.14751	11
peanut	-0.11527	10	butter	0.09349	3
tamarind	-0.11284	12	cheese	0.08038	1
tomato	-0.10687	8	coconut	0.05239	22
black bean	-0.09923	6	sesame seed	0.04636	6
black pepper	-0.09723	16	cream	0.04274	2
cinnamon	-0.08889	21	cocoa	0.04255	1
coriander	-0.08271	30	rice	0.03092	11
**Mughlai**					
**Ingredients contributing to negative food pairing**	**χ value**	**Frequency of occurrence**	**Ingredients contributing to positive food pairing**	**χ value**	**Frequency of occurrence**
ginger	-0.22264	20	milk	0.95554	71
garam masala	-0.22203	38	rice	0.46744	9
clove	-0.1727	42	bread	0.16189	12
cinnamon	-0.15605	33	grape	0.16132	3
tomato	-0.13042	21	mango	0.14838	11
ginger garlic paste	-0.10488	22	lemon	0.14672	8
green bell pepper	-0.10483	33	chaat masala	0.13532	13
cayenne	-0.09472	70	honey	0.12645	3
coriander	-0.07582	38	cream	0.10899	38
onion	-0.0696	29	soybean	0.08769	4
**Punjabi**					
**Ingredients contributing to negative food pairing**	**χ value**	**Frequency of occurrence**	**Ingredients contributing to positive food pairing**	**χ value**	**Frequency of occurrence**
garam masala	-0.18891	251	milk	0.16846	137
green bell pepper	-0.14559	301	bread	0.12552	60
cayenne	-0.1208	496	butter	0.10934	87
tomato	-0.10311	137	cheese	0.09834	7
mango	-0.10147	120	corn	0.05484	34
ginger garlic paste	-0.09551	110	lemon	0.0488	80
ginger	-0.08621	82	cottage cheese	0.03844	128
coriander	-0.08364	243	grape	0.03832	4
cinnamon	-0.06514	84	honey	0.03591	11
clove	-0.05827	86	olive	0.03388	16
**Rajasthani**					
**Ingredients contributing to negative food pairing**	**χ value**	**Frequency of occurrence**	**Ingredients contributing to positive food pairing**	**χ value**	**Frequency of occurrence**
garam masala	-0.13817	15	ginger	0.21659	3
coriander	-0.0901	35	mango	0.15163	21
clove	-0.07852	16	milk	0.14564	21
cumin	-0.07138	55	corn	0.09148	2
cinnamon	-0.05325	9	tamarind	0.07795	4
coriander cumin seeds powder	-0.04782	4	cardamom	0.03735	31
asafoetida	-0.03663	40	butter	0.03672	2
cayenne	-0.03646	80	lemon	0.02806	3
potato	-0.03488	3	bread	0.02767	2
black pepper	-0.03262	9	green bell pepper	0.02621	33
**South Indian**					

**Ingredients contributing to negative food pairing**	**χ value**	**Frequency of occurrence**	**Ingredients contributing to positive food pairing**	**χ value**	**Frequency of occurrence**
tamarind	-0.13638	87	rice	0.43068	119
tomato	-0.11714	51	garam masala	0.25363	24
green bell pepper	-0.11087	144	butter	0.19469	16
cayenne	-0.09829	238	black bean	0.1833	150
coriander	-0.06636	73	coconut	0.17749	68
curry leaf	-0.05268	196	mung bean	0.13281	34
peanut	-0.05027	16	milk	0.13233	26
ginger	-0.04228	24	cardamom	0.06319	46
lemon	-0.03363	20	soybean	0.04396	8
cumin	-0.03177	135	onion	0.0302	72

Table 1: Details of top 10 ingredients contributing to positive and negative food pairing in each of the regional cuisines.
